# Systems analysis and improvement approach to optimize outpatient mental health treatment cascades in Mozambique (SAIA-MH): study protocol for a cluster randomized trial

**DOI:** 10.1186/s13012-022-01213-8

**Published:** 2022-06-06

**Authors:** Vasco F. J. Cumbe, Alberto Gabriel Muanido, Morgan Turner, Isaias Ramiro, Kenneth Sherr, Bryan J. Weiner, Brian P. Flaherty, Monisha Sharma, Flávia Faduque, Ernesto Rodrigo Xerinda, Bradley H. Wagenaar

**Affiliations:** 1 Provincial Health Directorate, Sofala Province, Ministry of Health, Beira, Mozambique; 2 Faculty of Medicine, Eduardo Mondlane University, Maputo, Mozambique; 3 Department of Psychiatry, Beira Central Hospital, Beira, Mozambique; 4 Mozambican Health Committee, Beira, Mozambique; 5 Department of Global Health, University of Washington, Seattle, WA USA; 6 Department of Epidemiology, University of Washington, Seattle, WA USA; 7 Department of Industrial & Systems Engineering, University of Washington, Seattle, WA USA; 8 Department of Psychology, University of Washington, Seattle, WA USA; 9 Provincial Health Directorate, Manica Province, Ministry of Health, Chimoio, Mozambique

**Keywords:** Global mental health, Mozambique, Systems Analysis and Improvement Approach (SAIA), Optimization of care cascades, Process mapping, Continuous quality improvement, Primary mental healthcare, Task-sharing, Cluster randomized trial, Systems engineering

## Abstract

**Background:**

Significant investments are being made to close the mental health (MH) treatment gap, which often exceeds 90% in many low- and middle-income countries (LMICs). However, limited attention has been paid to patient quality of care in nascent and evolving LMIC MH systems. In system assessments across sub-Saharan Africa, MH loss-to-follow-up often exceeds 50% and sub-optimal medication adherence often exceeds 60%. This study aims to fill a gap of evidence-based implementation strategies targeting the optimization of MH treatment cascades in LMICs by testing a low-cost multicomponent implementation strategy integrated into routine government MH care in Mozambique.

**Methods:**

Using a cluster-randomized trial design, 16 clinics (8 intervention and 8 control) providing primary MH care will be randomized to the Systems Analysis and Improvement Approach for Mental Health (SAIA-MH) or an attentional placebo control. SAIA-MH is a multicomponent implementation strategy blending external facilitation, clinical consultation, and provider team meetings with system-engineering tools in an overall continuous quality improvement framework. Following a 6-month baseline period, intervention facilities will implement the SAIA-MH strategy for a 2-year intensive implementation period, followed by a 1-year sustainment phase. Primary outcomes will be the proportion of all patients diagnosed with a MH condition and receiving pharmaceutical-based treatment who achieve functional improvement, adherence to medication, and retention in MH care. The Consolidated Framework for Implementation Research (CFIR) will be used to assess determinants of implementation success. Specific Aim 1b will include the evaluation of mechanisms of the SAIA-MH strategy using longitudinal structural equation modeling as well as specific aim 2 estimating cost and cost-effectiveness of scaling-up SAIA-MH in Mozambique to provincial and national levels.

**Discussion:**

This study is innovative in being the first, to our knowledge, to test a multicomponent implementation strategy for MH care cascade optimization in LMICs. By design, SAIA-MH is a low-cost strategy to generate contextually relevant solutions to barriers to effective primary MH care, and thus focuses on system improvements that can be sustained over the long term. Since SAIA-MH is integrated into routine government MH service delivery, this pragmatic trial has the potential to inform potential SAIA-MH scale-up in Mozambique and other similar LMICs.

**Trial registration:**

ClinicalTrials.gov; NCT05103033; 11/2/2021.

Contributions to the literature

- This study will be one of the first in sub-Saharan Africa to rigorously test the effectiveness of an implementation strategy for optimizing the mental health treatment cascade in routine Ministry of Health primary healthcare clinics.
- This study also is one of the first to include the quantitative evaluation of hypothesized mechanisms of implementation strategy effect as part of the evaluation approach.
- If effective, the Systems Analysis and Improvement Approach for Mental Health (SAIA-MH) has a large potential to serve as a low-cost strategy to generate contextually relevant solutions to barriers to effective primary mental healthcare across low- and middle-income countries (LMICs).

## Background

Mental disorders are the leading cause of disability worldwide. In Mozambique, mental disorders account for 16.5% of years lived with disability (YLD) [1]. The treatment gap for mental disorders exceeds 90% in many low- and middle-income countries (LMICs) [2–4], in part due to a shortage of 1.2 million mental health (MH) workers globally [5]. To address this gap, academic and policy leaders have advocated for investments in rapid training programs for lower-level MH providers [6–12]. These investments are reaping benefits: since 2011 the number of MH nurses globally has increased by 35% [13].

**Fig. 1:**
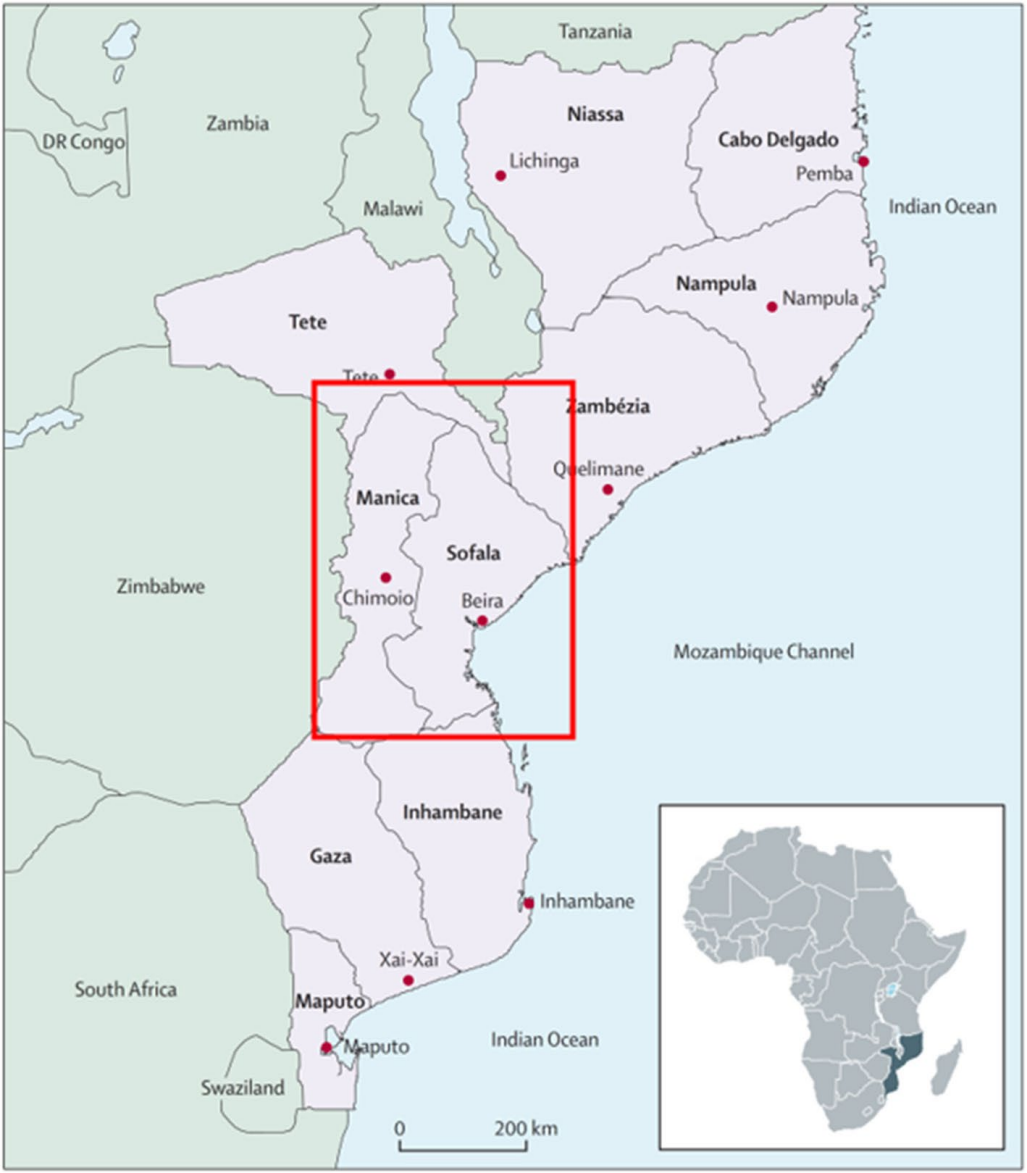
Mozambique with focal provinces of Sofala and Manica outlined in red. Figure sourced from Fernandes QF et al. (2014) [19]

Task-shared providers can deliver effective, evidence-based treatments for mental disorders in LMICs, and Mozambique has been a regional leader in MH task-sharing since 1996. It has been > 15 years since the first trials showed that MH treatments led by task-shared providers in LMICs could effectively treat mental disorders [14–16]. The 2016 *Disease Control Priorities* declared that task-shared outpatient treatment of common mental disorders in LMICs is cost-effective [17]. Mozambique has successfully scaled-up task-shared MH care despite only 0.16% of the health budget allocated to MH. The majority of MH care is provided by psychiatric technicians who complete a 2-year training program after acquiring at least a 10th-grade education [18]. The number of technicians working nationally increased from 66 in 2010 to 305 in 2019, achieving 100% coverage of at least one technician per district in 2014. In the province of Sofala (Fig. 1), increased coverage of technicians resulted in an 100% increase in outpatient MH consultations from 2012 to 2014 [20].

With most global investment focused on scaling-up task-sharing to close the MH treatment gap, less attention has been paid to implementation strategies that can assess and improve the quality of care delivered by existing providers with limited training, resources, and supervision. For example, in system assessments across Southern Africa, diagnosis was not recorded in 44% of cases, there was low awareness of non-psychotic mental illness, psychiatric history was inadequate in 89% of cases, inappropriate polypharmacy was prescribed in 88% of cases, and loss-to-follow-up (LTFU) exceeded 40% while sub-optimal medication adherence exceeded 60% [21–24]. Task-shared outpatient management of common mental disorders in Mozambique has shown LTFU rates > 50%, sub-optimal medication adherence > 80%, and < 30% of patients achieving functional improvement [25]. Similar patterns of suboptimal adherence, LTFU, and improvement outcomes have been reported in Nepal, Guinea-Bissau, and Tanzania [24–26]. There is an urgent need for strategies to optimize the task-shared MH treatment cascade. Until this need is met, the health effects of scaling-up task-shared MH care will be, at best, limited, and at worst, harmful in some settings.

The MH treatment cascade is a model that outlines the sequential, linked treatment steps that people with mental illness must frequently navigate, from initial diagnosis to symptom/functional improvement. Quality problems in one step of a treatment cascade can have non-linear and compounding impacts across the larger complex care system. Implementation strategies focused on only one step in a cascade can potentially contribute to unintended system bottlenecks and quality of care issues. By contrast, the *“Systems Analysis and Improvement Approach (SAIA)”* is a multicomponent implementation strategy focused on optimizing treatment cascades in their entirety. SAIA blends facilitation, ongoing clinical consultation, and routine service provider team implementation meetings with systems-engineering tools in a 5-step approach specifically developed for task-shared providers. The five steps of SAIA include (1) cascade analysis to visualize treatment cascade drop-offs and prioritize areas for system improvements; (2) process mapping to identify modifiable facility-level bottlenecks; (3) identification and implementation of modifications to improve system performance; (4) assessment of modification effects on the cascade; and (5) repeated analysis and improvement cycles.

A previous 9-month cluster RCT established the effectiveness of SAIA for HIV treatment cascade improvement across Mozambique, Cote-D’Ivoire, and Kenya, showing a 3.3-fold greater improvement in ART uptake for HIV-infected pregnant women (13.3% vs. 4.1% increase), and a 17-fold greater improvement in early infant diagnosis for HIV-exposed infants (11.6% vs. 0.7% increase) [27]. Intervention facilities tested an average of 9 system modifications over the 9-month intervention, and over 80% of those “micro-changes” were considered successful by facility personnel. However, no effectiveness data exist on SAIA applied to non-HIV treatment cascades such as task-shared MH care. Preliminary data suggest that applying SAIA to MH treatment cascade optimization is feasible, acceptable, and can result in clinically significant treatment cascade improvements. Five months of pilot SAIA-MH implementation resulted in a 1.5-times higher odds of medication adherence (aOR 1.5; CI 1.2, 1.9) and a 3.7-times higher odds of functional improvement (aOR 3.7; CI 2.5, 5.4) [25]. Systems changes tested included service re-organization, new interventions, patient education, and improving data and its use. The SAIA-MH strategy was feasible: all teams adopted the strategy and conducted 5 optimization cycles during the 5-month period. These pilot data suggest that SAIA-MH is a promising strategy for task-shared MH systems improvement globally.

The overall goal of the present study is to evaluate the real-world effectiveness of the multi-component SAIA-MH implementation strategy to optimize outpatient mental health services in Mozambique. Specific aim 1a will assess effectiveness using a 3-year parallel cluster randomized trial across 8 intervention and 8 attentional control facilities. Specific aim 1b will test causal pathway models to analyze mechanisms of action for effects (or non-effects) of the SAIA-MH implementation strategy. Specific aim 2 will focus on estimating the cost and cost-effectiveness of scaling-up SAIA-MH in Mozambique. This study is innovative in extending an evidence-based implementation strategy from the field of HIV to MH treatment, while also being the first to test not only whether the SAIA implementation strategy works—but how it works—through testing causal pathway models. If effective, the SAIA-MH implementation strategy has large potential to be rapidly scaled-up to decrease gaps in task-shared MH systems globally.

## Methods

### Systems Analysis and Improvement Approach for Mental Health (SAIA-MH) Implementation Strategy Specification

The SAIA-MH implementation strategy builds off continuous quality improvement [28] (CQI) [29] influenced by the Donabedian model [30], a framework for assessing quality of care based on interconnected components of structure, process, and outcomes. *Structure* describes attributes of the setting in which a provider delivers care (organizational structure, along with material/human resources). *Process* describes what is done to the patient as that individual receives services. Both can be measured and manipulated. Health *outcomes* are a function of the structure and process and changes made to them. The SAIA strategy is a multicomponent package of distinct implementation strategies [31] targeting a variety of individual, organizational, and contextual factors that foster or impede implementation success [32–34] (Table 1). In addition, SAIA uses systems-engineering [35] principles to support task-shared workers to diagnose and prioritize problems; evaluate decision options using optimization; and recommend, implement, and evaluate actions considering the treatment cascade as a whole. By design, the SAIA model guides workers to generate ideas to address local problems in the treatment cascade, resulting in a high likelihood that proposed changes are appropriate for the local context and acceptable to the implementing team. Thus, the SAIA model focuses on increasing the chances of system changes being sustained over the long term.

**Table 1:** Distinct SAIA-MH implementation strategies coded to the Expert Recommendations for Implementing Change (ERIC) framework [31]

Distinct SAIA-MH Implementation Strategy coded to ERIC	Individual, organizational, or contextual barrier(s) addressed
External facilitation	Lack of knowledge of quality improvement and SAIA-MH implementation strategy
Provide ongoing clinical consultation	Clinical knowledge gaps; gaps in clinical evaluation and reporting
Organize service provider implementation team meetings	Limited teamwork: lower-level providers afraid to innovate without approval; siloing of services and providers; issues with role clarity
Step 1: Cascade analysis (ERIC: Audit and feedback; Model and simulate change; facilitate relay of clinical data to providers)	Lack of knowledge of problems; no data for prioritization; data only collected/reported but limited feedback; limited accountability
Step 2: Process mapping (ERIC: Conduct local needs assessment; assess readiness for change and identify barriers/facilitators; conduct local consensus discussions)	Lack of consensus on current system; limited teamwork; limited discussion on full system, goals, barriers, and facilitators; hard to conceptualize potential modifications
Steps 3–5: Conduct cyclical tests of change (ERIC: Conduct cyclical small tests of change; tailor strategies; develop a formal implementation blueprint; purposely reexamine the implementation)	Limited culture of quality; providers rigidly follow guidelines with no ability to innovate and improve

**Fig. 2:**
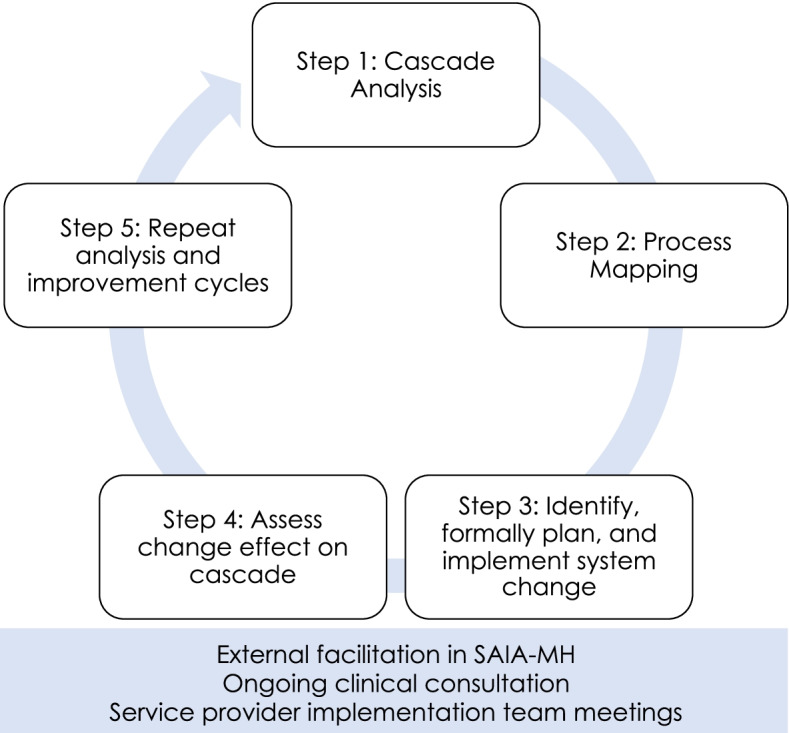
Systems Analysis and Improvement Approach for Mental Health (SAIA-MH) implementation strategy processes

The SAIA-MH implementation strategy blends external facilitation [36], ongoing clinical consultation, and service provider implementation team meetings with system-engineering tools in an overall CQI framework [28, 37] (Table 1; Fig. 2). External experts in the SAIA implementation strategy partner with respected, knowledgeable local district/provincial management in the health system. These local leaders work with the external SAIA team to contextualize and adapt the SAIA strategy to the specific context. Such adaptation has been performed as part of the original HIV-focused SAIA trial [29], as well as the SAIA-MH pilot study [25]. This included (1) specifying a MH “care cascade” to be optimized that was both feasible to populate with routine health systems data as well as relevant in the Mozambican public-sector context; (2) developing enhanced MH clinical registries and patient tracking tools to allow population of the MH care cascade; (3) finalizing the Mozambican mental health cascade analysis tool (MHCAT) used to provide a rapid systems-level view of drop-offs along the MH care cascade with an optimization function to help prioritize which step in the care cascade is weakest. Following contextualization, clinic directors, managers, and all staff directly involved in MH care delivery form a facility-level MH systems improvement team and attend a 1-week training co-led by the local district/provincial management and external SAIA-MH experts. Afterwards, the external SAIA-MH team and local management provide facilitation with facility-level system improvement teams, guiding them through the monthly iterative 5-step SAIA-MH implementation strategy process [29]. This process gives facility teams a systems-wide view of the treatment cascade performance within their health facility, guides them in collaboratively understanding their current system, helps them to *diagnose* problems, *prioritize* areas for improvement, and then *recommend, implement, and evaluate* improvements [35].

**Fig. 3:**
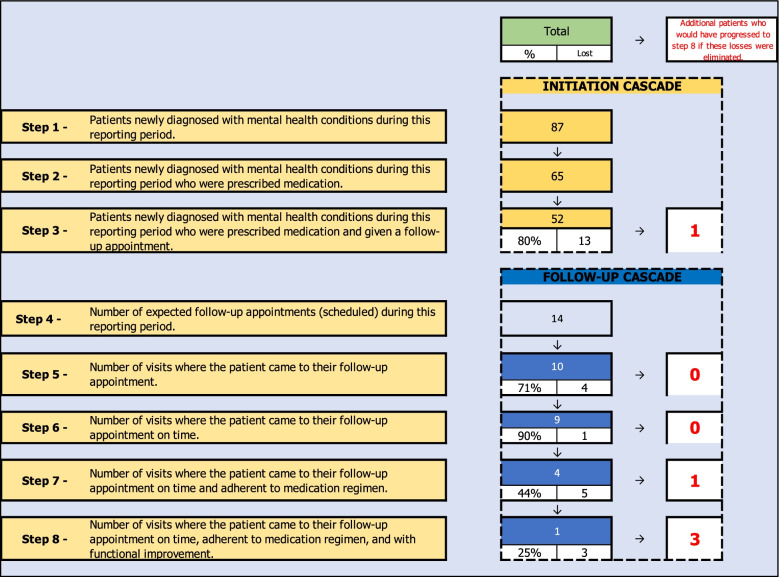
Mental health cascade analysis tool (MHCAT)

The 5 steps of SAIA-MH are the following:

- Step 1: Cascade analysis—understand MH cascade performance and identify priority areas for systems improvement [38, 39]. The MHCAT (Fig. 3) uses routine facility-level data to provide a systems-level view of the interdependent components determining care delivery. As an analytic tool, the MHCAT provides MH staff with a view of the weakest areas of the current treatment cascade and aids teams in prioritizing targets for systems improvement. The MHCAT, along with run charts and process indicators, will also help facility teams evaluate the impact of small tests of systems change in later steps.
- Step 2: Process mapping—diagnose facility-level modifiable bottlenecks and gain consensus on current system. Enabling facility-level staff to diagnose and gain consensus on bottlenecks to address in their MH systems is essential to defining system improvements to test. SAIA-MH applies sequential process mapping, coupled with workflow observation, to build teamwork and consensus on existing system structure and guide discussion on opportunities for improvements.
- Step 3: Identify, formally plan, and implement system change. After diagnosing modifiable system barriers, facility teams identify a simple, specific change to improve performance within the prioritized cascade step. Selected system changes should be feasible to implement, be within the scope of influence of facility teams, and be expected to lead to rapid, substantial improvement. Facilitation is provided to help teams identify improvements. Facility teams make a formal implementation plan to ensure consensus and clarify operational design/roles. Steps 3–5 borrow heavily from CQI.
- Step 4: Monitor changes in routine performance. Facility teams monitor changes in a selected process indicator, as well as track patients progressing through targeted steps using run charts. These indicators capture large, rapid improvements.
- Step 5: Repeat analysis and improvement cycles. Systems-engineering [35] is by definition iterative given that system innovations must respond to evolving, contextually specific barriers. Facility teams repeat steps 1–5 during each monthly cycle to identify new approaches to modify identified barriers, or if the first cycle was successful, focus on identifying new priority barriers.

### Study aims

The present project proposes to conduct a 3-year parallel cluster RCT across 8 intervention and 8 attentional control facilities to evaluate the real-world effectiveness of the SAIA-MH implementation strategy on mental health functional improvement (primary) and patient retention in care/medication adherence (secondary). Specific aim 1b will include testing causal pathway models to analyze mechanisms of action of the SAIA-MH strategy using longitudinal structural equation modeling as well as specific aim 2 estimating the incremental cost and cost-effectiveness of scaling-up SAIA-MH in Mozambique using micro-costing, time-and-motion observation, and a Markov model parametrized with cost and outcome data from the SAIA-MH cluster RCT.

### Overview of study design

We will implement a 3-year cluster RCT, randomizing 16 clinics currently providing outpatient mental health care to treatment (8 clinics) and attentional placebo control (8 clinics). We will include a 6-month baseline period prior to randomization, followed by a 2-year intervention or attentional placebo control period, and an additional 1-year sustainment phase (Table 2). During the 6-month baseline phase, all clinics will receive enhanced patient registries and patient tracking tools as used in the pilot study [25]. All facilities will receive monthly in-facility mentorship on the new data tools, allowing for examination of baseline cascade performance. During these monthly visits, data from the patient registries and tracking tools will be entered into the CommCare database by study staff. Following the baseline period, clinics will be randomized to the SAIA-MH implementation strategy or attentional placebo control.

**Table 2:**
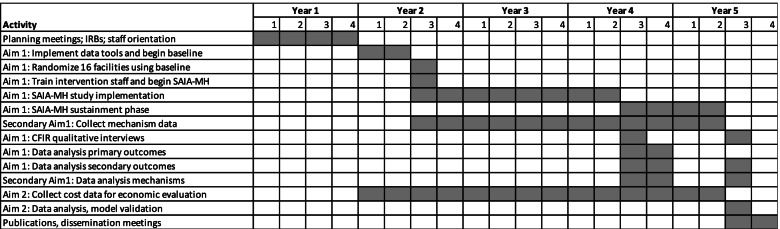
Research project activities and timeline

Those receiving SAIA-MH will attend a 1-week in-person training for facility improvement teams. Facility-level teams will be determined by facility staff but will need to include a minimum of one psychiatric technician, one psychologist, and the clinic manager/director who oversees MH delivery. Following the 1-week in-person training, SAIA-MH standard operating procedures will be implemented, including: (1) structured external facilitation and clinical consultation following tablet-based guides used in pilot study (1× per week first month; 2× per month for next 2 months; 1× per month for remainder); (2) facilitation in the 5-step SAIA-MH improvement process, including: (3) cascade analysis and prioritization using the MHCAT; (4) process mapping for bottleneck identification; (5) defining system changes/developing implementation plans; and (6) evaluating and revising system changes. As in the original HIV-focused SAIA trial [29], facilities will be able to request reimbursement of up to $50/month for essential supplies needed to test systems changes. During these regular facilitation visits, clinic staff who are participating in the intervention will complete questionnaires designed to measure moderators, preconditions, and hypothesized mechanisms of SAIA-MH. In-facility facilitation and clinical consultation early in SAIA-MH implementation is intensive and has proven critical to helping teams begin to think from a “systems-engineering perspective” [35]. SAIA-MH protocols suggest cycles occur monthly, corresponding with monthly MHCAT systems analysis data, thus, we anticipate that facilities will complete 24 systems improvement cycles during the 2-year intensive implementation phase and 12 during the sustainment phase.

We will employ an attentional placebo control design whereby control facilities will mimic activities of the intervention group in time and contacts, but without the “active ingredient” of the SAIA-MH implementation strategy [40]. This design protects against key threats to internal validity, such as the Hawthorne effect [40], testing effects [41], and generally accounts for non-specific effects of SAIA-MH activities in a similar way that a placebo is used to account for expectancy effects in a drug trial [40]. Thus, facilities randomized to attentional placebo control will attend a 1-week in-person training for facility learning collaboratives determined by facility staff, including the same minimum staff above for SAIA-MH. This training will focus on a review of the enhanced patient registries and patient tracking tools, as well as discuss ethics, mental health stigma, and burnout for mental health professionals. Following the 1-week in person training, attentional placebo control facilities will receive monthly in-clinic facilitation following the same schedule as SAIA-MH paired with monthly data collection on moderator, precondition, and hypothesized mechanisms. External facilitation will focus on review of the use of the enhanced patient registries and patient tracking tools. As the SAIA-MH implementation strategy occurs at the systems level in public sector clinics in Mozambique, there will be no restrictions placed on concomitant care. Any observed external changes in the health system suspected of influencing study outcomes will be noted in final published materials. Important protocol modifications will be communicated to both ethics committees at the University of Washington and in Mozambique and a communication plan with stakeholders developed under their simultaneous guidance.

During the 1-year sustainment phase, management of data collection to produce the MHCAT will transfer fully to the provincial statistics office and SAIA-MH facilitation will be done only by local management to examine effectiveness and sustainability under fully routine conditions. Provincial authorities currently collect patient-level MH data monthly to report diagnostic and outcome data within the Ministry of Health system. Primary effectiveness analyses of the SAIA-MH implementation strategy will occur at the end of the 2-year intervention period. This project will partner with Ministry of Health staff and support the automation of existing monthly reports, with the simultaneous creation of the MHCAT cascade analysis for data feedback to the facility level.

### Study setting

Manica and Sofala Provinces (population: approximately 4 million; Fig. 1) were selected because of the deep relationship between investigators and the local Ministry of Health as well as the absence of existing structural interventions for MH systems improvement. The Mozambican Ministry of Health has been a leader in sub-Saharan Africa in scaling-up task-shared outpatient mental healthcare led by mid-level specialist providers called psychiatric technicians. These providers can treat all categories of mental, neurological, and substance-use disorders using both pharmacological and psychosocial evidence-based interventions. In 2014, Mozambique achieved its goal of having at least one psychiatric technician providing outpatient mental healthcare in each of the 128 districts of Mozambique [18]. However, existing evidence suggests that the quality of mental healthcare in Mozambique remains low [42] due to limited financial and human resources, few opportunities for supportive supervision, training, and re-training [18], stock-outs of essential mental health medications [43], and mental health stigma [44] among other challenges [25]. In central Mozambique, over 98% of formal health services are offered through the public sector [45], improving the potential for population-level impacts for supply-side systems interventions.

### Study outcomes

Primary SAIA-MH study outcomes will be individual-level patient functional improvement (primary), medication adherence (secondary), and retention in care (secondary) (Table 3). Patient functional improvement was selected as the primary trial outcome because it had the largest relative increase in the pilot study (aOR = 3.7, 8.9% increase, improvement from 4.2 to 13.1%) and it is the ultimate goal of MH treatment as the last step in the care cascade; therefore, improvements in upstream cascade steps and process/quality indicators should be expected to also improve functional improvement. The WHODAS 2.0 for primary outcome measurement was developed by the World Health Organization (WHO) specifically to allow cross-cultural disability measurement across settings and patient populations [46]. It has good reliability, item-response characteristics, and a robust factor structure that has been replicated across sub-Saharan Africa [46–49]. In the original WHO validation study across 19 countries (9 LMICs) the test-retest reliability had an intra-class coefficient of 0.98, the total Cronbach’s alpha (*n* = 1565 across 19 countries) was 0.98 and also 0.98 for MH patients [46, 49]. The WHODAS 2.0 has been used extensively in LMICs [46–50], shows good performance among diverse MH diagnoses [51–53], has been applied successfully in Mozambique in previous studies [50], including our pilot study, and has been extensively applied in LMICs (including Sub-Saharan Africa) to evaluate functional improvement in pragmatic MH trials [54–59]. Primary study outcomes will be sourced from a census of individual-level patient records from outpatient routine care, which will also be used to populate the MHCAT tool.

**Table 3:** Primary study outcome definitions. All patient and facility-level outcomes refer to all patients attended in outpatient mental health services in target health facilities

Outcome	Indicator	Definition
**Patient-level clinical**	Primary: functional improvement	Patients w/ ≥ 1 follow-up w/ score ≤ 10 or ≥ 50% reduction in baseline WHODAS 2.0
	Secondary: medication adherence	Patients returning for follow-up visit not missing a dose (patient report and pill counts)
	Secondary: retention in care	Patients attending scheduled follow-up appointments; and attending those appointments on time.
**Patient-level process and quality**	Vitals recorded	Patients with height, weight, and blood pressure recorded
**Facility-level process and quality**	New patient diagnosis	# of new MH patients diagnosed
	Treatment initiation	# of new MH patients starting medication
	Total patient load	# of MH patients with ≥ 1 follow-up visits in last 3 months
**Implementation outcomes**	Acceptability	% of facility staff reporting satisfaction with SAIA-MH and various strategy components
	Adoption	% of trained facility staff engaging in ≥ 1 implementation plan in first 3 months; # of SAIA-MH cycles completed in first 6 months
	Fidelity	% of teams following 5-step SAIA-MH process
	Cost	See economic evaluation section
	Penetration	% of trained facility staff engaging in ≥ 1 implementation plan during 1-year sustainment phase
	Sustainability	# of SAIA-MH cycles completed during sustainment phase; facility staff intent-to-continue-use; clinical, process, and quality outcome trends during sustainment

### Sampling and randomization procedures

Eligible facilities are public-sector Ministry of Health clinics naïve to SAIA-MH, with ≥ 100 average annual outpatient MH consultations from 2019 to 2020 and the presence of a minimum of one psychiatric technician and one psychologist. For feasibility regarding project implementation in an area with challenges during rainy season, inclusion criteria for facilities included being within a 3-h one-way drive from Chimoio City, Manica Province, or Beira City, Sofala Province. Last, quaternary or provincial hospitals were excluded due to the complexity of mental health workflows across specialty services. Given these criteria, there were 9 eligible facilities in Manica Province and 8 eligible facilities in Sofala Province (Table 5). Eligible facilities will be allocated 1:1 to intervention or control using constrained randomization to maximally balance province, level of health facility, MH human resources, number of annual outpatient MH consultations, and baseline cascade performance. Baseline cascade performance will be facility-level achievement of patient functional improvement during the 6-month baseline period (Table 3). There will be no blinding of facility assignment at the level of providers, managers, or investigators as it is not feasible given SAIA-MH implementation procedures. The only blinding will occur at the level of the outcome assessor (statistical analyst). Randomization will be conducted in Stata 16.0 using the ccrand command for covariate-constrained randomization in cluster-randomized trials [60]. The allocation sequence will be generated by the senior author (BHW) and enrollment—including informed consent—led by technical implementation leads in Mozambique (VFJC and AM).

### Data collection and management

**Fig. 4:**
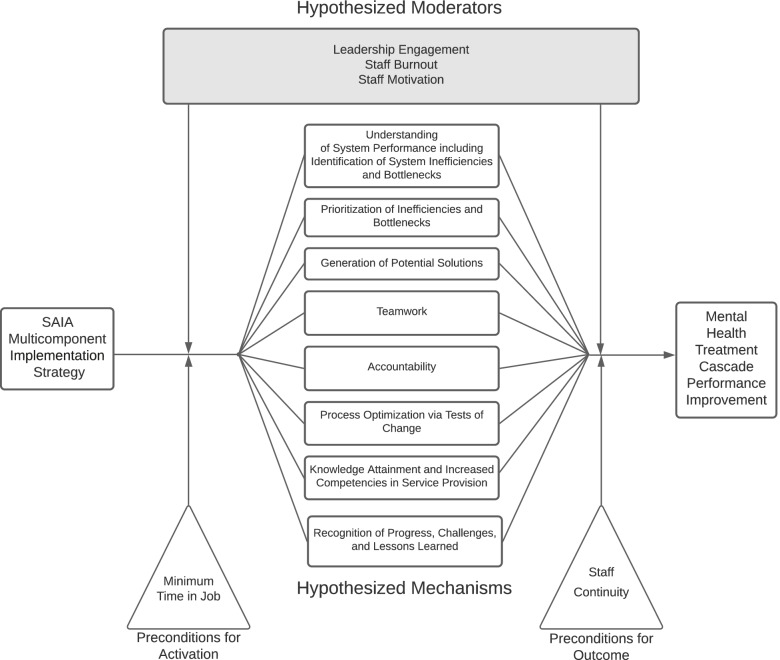
Causal pathway model of hypothesized mechanisms of SAIA-MH effects

Patient- and facility-level process and quality information (Table 3) will be abstracted into a separate CommCare [61] database by study team staff. CommCare is an android-based platform to support mobile data collection specifically in low-resource settings globally. De-identified aggregate patient and visit-level data from CommCare will be directly linked to Tableau [62] data visualization software to visualize facility-level MHCAT results in real-time. MHCAT visualizations will be shared with each facility during the scheduled supervision and implementation team meetings. Separate CommCare forms will be used to track action planning, facilitation activities, mechanisms, and costs of SAIA-MH implementation. These supervision forms will be filled out by the study team during each supervision visit. Thus, primary study outcomes, implementation outcomes, and other process measures will be sourced from: (1) CommCare patient-level data collection and supervision forms/questionnaires; and (2) qualitative CFIR interviews. Monthly data will include longitudinal documentation of changes in mechanisms of effects for all 16 facilities (Fig. 4), fidelity to the SAIA-MH model (intervention only), and barriers/facilitators to implementation. During the sustainment phase, collection, and management of the CommCare and Tableau data will transition fully to the provincial statistics offices of Sofala and Manica provinces. Research and clinical staff will be trained to identify potential adverse events and instructed to report them immediately to the senior author (BHW), implementation leads (VFJC and AM) and the country director for implementation activities in Mozambique (IR). Adverse events will be reported under the schedule expected by the University of Washington ethics committee.

### Primary statistical analyses

Individual-level primary, secondary, and process/quality outcome data will be analyzed using generalized linear mixed models using random effects at the facility and individual level. Family and link functions will be determined based on outcome distributions, although *a priori* specification will utilize binomial family and logit links to evaluate the odds of progressing through the MH care cascade. Absolute and percentage changes in WHODAS 2.0 scores between patients in intervention and control facilities will also be computed. Primary analyses will compare changes from the 6-month baseline to the 2-year intensive implementation period for intervention versus control facilities. Analyses will be repeated comparing effects over the sustainability phase to both baseline and implementation phases. Patient (age, sex, HIV/TB diagnosis, suicidal ideation) and health-facility adjustment factors (patient load, staff turnover or absence, distance from district/provincial headquarters) will be considered for inclusion. Sex as a biological variable will be assessed using pre-specified interaction sub-analyses to examine differential SAIA-MH effects for females vs. males. Dose-response analyses by levels of fidelity to protocol will also be examined. We will also conduct a controlled, segmented time-series analysis [63, 64] that incorporates monthly facility-level estimates from the entire 42-month study period (segmented into 6-month pre-intervention; 24-month intervention; and 12-month sustainment periods). This secondary analysis will allow assessment of SAIA-MH on number of new diagnoses, treatment initiation, total patient load, as well all other MH cascade outcomes, addressing both serial/intra-cluster correlation, and temporal patterns in SAIA-MH effectiveness. These facility-level time-series analyses will also potentially allow exploratory examination of the effects of specific systems modifications. Modifications found to have large effects will be documented as part of identifying best practices for MH care cascade improvement. All statistical analyses will be conducted blinded to study arm (intervention vs. control).

### Primary study power assessment

In the pilot, SAIA-MH increased patient functional improvement with an adjusted odds ratio of 3.7 and an absolute increase of 8.9% (4.2% control; 13.1% intervention) [25]. The intra-cluster correlation coefficient using outpatient mental health data in the same setting is estimated at 0.26 [https://pophealthmetrics.biomedcentral.com/articles/10.1186/s12963-015-0043-3]. Selected clinics in Table 4 conducted an average of 774 outpatient MH visits per 6 months in 2019–2020. In the pilot, 40% of patients returned for follow-up within 60 days in control periods; thus, we estimate target clinics will see approximately 300 follow-up patients per 6 months. Using these assumptions, we will have ≥ 90% power to detect an increase as small as 2.2% in functional improvement (primary), 3.6% for adherence (secondary), and 4.9% for retention (secondary); (Table 5).

**Table 4:** Facility-level characteristics

Facility	Province	Mean annual outpatient mental health visits 2019–2020	Level	Number of psychiatric technicians	Number of psychologists	Minutes to drive from Provincial Capital
Nhamatanda	Sofala	4000	Rural hospital	2	3	90
Dondo Sede	Sofala	2885	Urban health center type A	1	3	40
Macurungo	Sofala	1588	Urban health center type A	1	1	15
Mafambisse	Sofala	1306	Rural health center type 1	1	1	30
Chingussura	Sofala	981	Urban health center type A	1	2	26
Mascarenhas	Sofala	752	Urban health center type B	1	2	16
Muxúngue	Sofala	1107	Rural hospital	1	1	180
Inhamizua	Sofala	664	Urban health center type B	1	1	30
Manica	Manica	3605	District hospital	2	2	1
Gondola	Manica	1314	District hospital	2	1	20
Catandica	Manica	983	District hospital	2	2	120
Sussundenga Sede	Manica	964	Rural health center type 1	1	2	60
Macate	Manica	466	Rural health center type 2	1	1	60
Nhamaonha	Manica	284	Urban health center type B	1	1	10
Vila Nova	Manica	195	Urban health center type A	1	1	15
Vanduzi	Manica	3675	Rural health center type 1	1	1	30

**Table 5:**
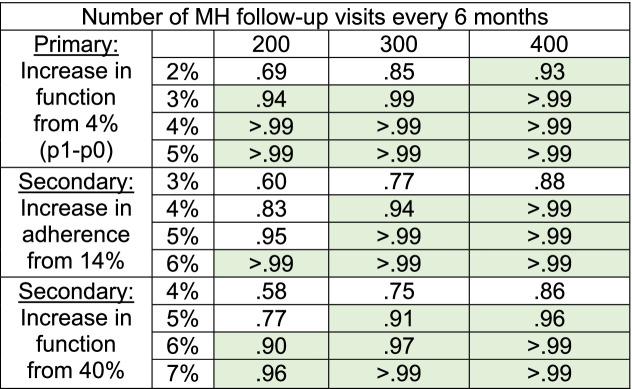
Power under various conditions. Green indicates power greater than or equal to 90%

### Qualitative data collection and analyses

At the end of the 2-year intensive implementation phase, we will conduct focus group discussions (FGDs) among each facility-level improvement team (*n* = 16) to collect in-depth qualitative data on CFIR constructs of interest (Table 6). Questions will be sourced from CFIR interview guides (http://cfirguide.org/), supplemented by targeted elicitation of satisfaction with tools/protocols, intent-to-continue-use, and deviations from SAIA protocols. FGDs will occur after primary quantitative analyses, with facilities classified as high, medium, or low performing in terms of fidelity to SAIA-MH as well as cascade improvements. By classifying clinics, we intend to uncover salient features and determinants of successful implementation. Following analyses of mechanisms of effect, we will also classify a sub-set of individual staff (*n* = 10–20) and facilities (*n* = 4–6) into groups who showed large improvements in causal mechanistic pathways and those with little improvement across targeted mechanisms. These staff will be targeted for additional in-depth-interviews (IDIs, individuals) or FGDs (facilities) organized around the CFIR and paired with targeted elicitation of qualitative explanatory data on barriers/facilitators to specific mechanisms of action. These data will help: (1) inform the need for tailoring of SAIA-MH for specific facility and individual-level contexts that may hinder or enhance mechanism activation; (2) understand other potential moderators, mediators, preconditions, or mechanisms not captured in current causal pathway models; and (3) further unpack the mechanistic reasons SAIA-MH may show effects in some settings, and for some individuals, and not others. To refine models and understand implementation mechanisms, processes, and determinants under routine conditions, both rounds of FGDs and IDIs will be repeated at the end of the sustainment phase. IDIs and FGDs will be conducted in Portuguese by an experienced facilitator accompanied by a note-taker, audio-recorded, and transcribed verbatim into Portuguese by trained Mozambican staff. Using existing CFIR codebooks as a guide, two researchers will use a stepwise, iterative process to review transcripts and identify key deductive themes (using select CFIR constructs, implementation outcome, and targeted mechanism themes) while allowing for flexibility of other inductive themes to emerge. Following iterative coding of transcripts, the team will convene with the PI to identify coding discrepancies with the coding process repeated until consensus is achieved.

**Table 6:**
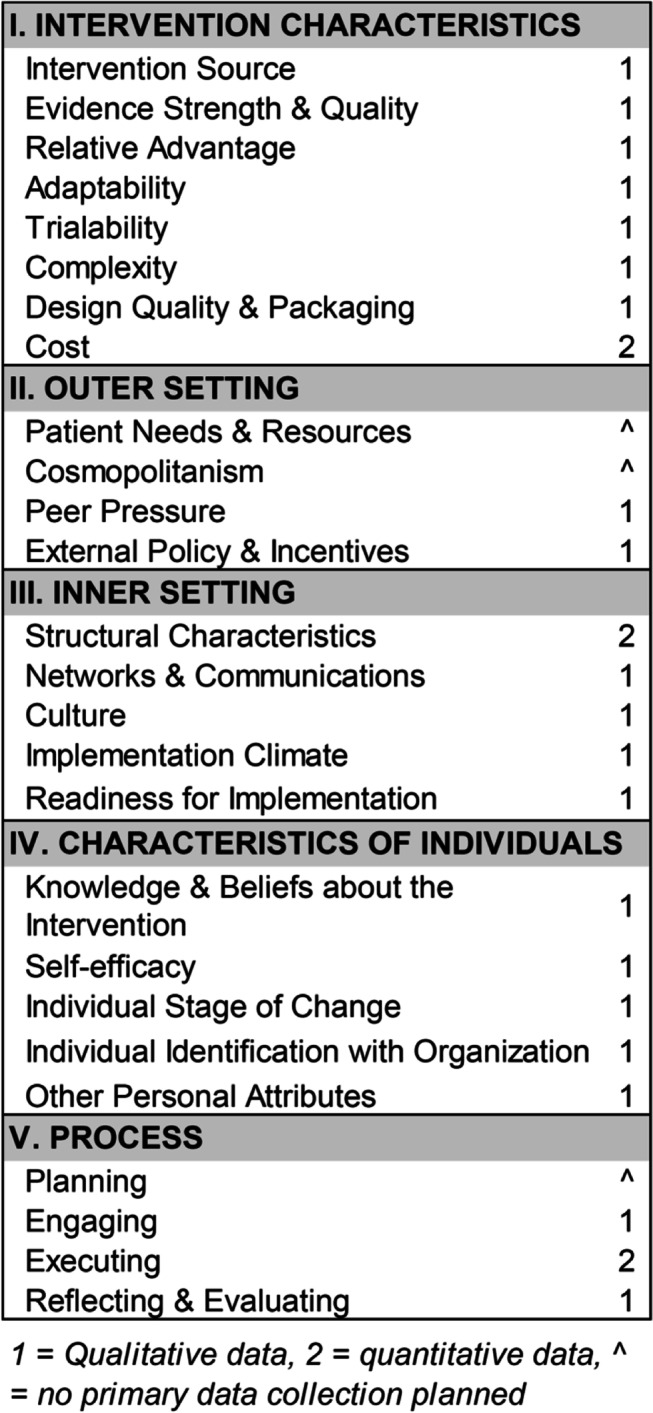
Consolidated Framework for Implementation Research (CFIR) constructs of interest

### SAIA-MH mechanisms assessment approach

Using theoretical bases informing SAIA implementation strategy development, a review of literature for similar distinct implementation strategies (audit and feedback [65–69], quality improvement [70–75], facilitation [76, 77]), and mixed-methods quantitative [27] and CFIR qualitative data [27] from the original HIV-focused SAIA trial and the SAIA-MH pilot study, we have developed an initial causal pathway model to test in the present study (Fig. 4). Each moderator, precondition, and hypothesized mechanism will be measured each month during baseline, intensive implementation, and sustainment periods across all 8 intervention and 8 attentional placebo control facilities during facility-level facilitation/supervision visits. For clarity, we hypothesize that attentional placebo controls will show no significant activation of SAIA-MH mechanisms but will account for key threats to validity such as testing, maturation, external shocks, and Hawthorne effects. Mechanisms will be assessed monthly at the individual provider level and then aggregated within facilities across time, while MH cascade performance will be sourced from individual patient CommCare records and then aggregated within facilities across time. Primary analyses of mechanisms will occur after the intensive phase (Table 2). Secondary analyses will occur at the end of the sustainment phase.

### SAIA-MH mechanisms assessment power

Overall, this study was powered to specific aim 1a examining mean treatment effects of SAIA-MH on MH treatment cascade performance. Given the paucity of literature on methods, theories, and frameworks underlying mechanisms of multicomponent implementation strategy effects, this aim will focus on an exhaustive examination in search of practically and clinically meaningful mechanism, moderator, and precondition effect estimates for further examination in subsequent research. The goal of this aim is to contribute to theory building to inform future studies specifically powered to test hypothesized causal pathway models. No confirmatory hypothesis testing driven by *p* value inference will be performed, as recommended when conducting exploratory studies by Leon (2011) [78]. That said, we will have a sufficient sample size to have stable effect estimates with reasonable precision based on our design including 6 baseline measurements and 24 implementation measurements among a minimum of 3 providers across all 16 facilities. Assuming an intra-cluster correlation coefficient of 0.25 [20], if a binary mechanism leads to a function improvement odds ratio of 1.5, we will have > 0.85 power to detect the indirect effect with variance of the mediator assumed to be 0.5. Continuous mechanisms will have appreciably higher power.

### SAIA-MH mechanisms assessment analyses

Our analytical plan will progress in 3 stages and across 3 separate patient-level outcomes: functional improvement (primary), medication adherence (secondary), and retention (secondary). Repeating the analytic process across 3 dependent variables provides built-in replication, allowing the identification of consistent effects (direct, indirect, and moderated). First, we will conduct univariate examination of hypothesized mechanism effects using multi-level longitudinal SEM accounting for multiple providers nested within facilities across time. The purpose of this will be to examine single main effect estimates for hypothesized mechanisms. For example, we will estimate a univariate multi-level longitudinal SEM of SAIA-MH strategy effect predicting changes in teamwork, then predicting MH treatment cascade outcomes (Fig. 4). We will conduct diagnostics, including assessing linearity of mechanism effects, model residual distributions, and variance accounted for in mechanisms and outcomes. We will then test potential moderators in univariate models, in search of those that strongly affect strength or direction of univariate effects. Moderators will be examined for their effect on SAIA-MH strategy activation of mechanisms (left side of Fig. 4) as well as mechanism effects on outcomes (right side of Fig. 4); preconditions will be tested alongside moderators. Second, we will include all mechanisms simultaneously in a multivariable longitudinal multi-level SEM. Including all mechanisms, even those showing no effects in univariate models accounts for potential suppressor effects [79]. Moderators that showed meaningful effects in univariate models will then be included in a final multivariable model. Final model diagnostics will be verified, and variance accounted for in mechanisms, as well as outcomes, will be reported. This multivariable model will allow examination of (1) mechanisms most affected by SAIA-MH (left side Fig. 4); (2) mechanisms that had an effect on outcomes (right side Fig. 4); and (3) the product of #1 and #2 path coefficients indicating the indirect effect of SAIA-MH on treatment outcomes operating through a given mechanism. Separating out paths #1 and #2 can inform improvement of the SAIA-MH strategy; for example, if a mechanism had a large effect on outcomes (path #2), although was only weakly activated by SAIA-MH (path #1), the SAIA-MH strategy could be modified to enhance activation of this potentially high-leverage mechanism. Findings from path analyses will be incorporated into targeted elicitation of explanatory qualitative information during the second round of CFIR interviews. Individuals/facilities will be classified into those showing large mechanism changes compared to those with little improvement.

### Costs

We will conduct activity-based costing and time and motion observation at both intervention and control clinics to estimate the incremental cost of implementing SAIA-MH. Throughout study implementation, we will estimate all costs that would be incurred by the Ministry of Health to administer the SAIA-MH intervention. These costs will be collected and tabulated using activity-based cost menus during start-up, intensive implementation, and sustainability periods. Costs include patient visits, medication, personnel, supervision, supplies, buildings and overhead, strategy delivery, facility support for systems changes, training, and equipment. Time-motion observations following protocols previously used by our group in Mozambique [80] will estimate personnel time needed for SAIA-MH tasks.

### Economic evaluation

We will develop a Markov cohort model using R software reflecting the natural history of MH disease progression and disability, and treatment, which has been used for previous cost-effectiveness analyses of MH interventions in LMICs [81–87]. The model will simulate a cohort with different MH conditions stratified by retention in care, medication adherence, and functional improvement. We will parameterize the model with cost and outcome data from the SAIA-MH cluster RCT and facility-level contextual data (patient loads and trends; human resources; facility types) and demographic data (gender; age distributions). In line with previous studies and economic guidelines [81, 88], costs and effectiveness will be discounted at 3% annually (varied from 0-5% in sensitivity analyses). Disability weights for DALYs will be obtained from the Global Burden of Disease 2017 [89]. Scale-up scenarios will model changes in patient retention, adherence, and functional improvement based on study results and varied in sensitivity analyses. Prior to conducting cost-effectiveness analyses, we will assess model validity/reliability by evaluating our model’s ability to accurately project trends in care cascade outcomes (retention, medication adherence, functional improvement).

We will simulate the health and economic impacts of scaling-up the SAIA-MH implementation strategy in Mozambique at district, provincial, and national levels. Health outcomes include effects on the outpatient mental health cascade (proportion retained, medication adherent, and with functional improvement). The model will estimate the effects of scale-up scenarios on: (1) retention and adherence in MH care by diagnosis; (2) functional improvement by diagnosis; and (3) Disability Adjusted Life Years (DALYs) [88] averted, cost per DALY averted, and budgetary impact from the payer (Ministry of Health) perspective. Different scenarios will examine rate and breadth of expansion (number/characteristics of future facilities/districts/provinces). We will estimate incremental costs, as well as treatment costs incurred and averted due to the implementation strategy.

We will project SAIA-MH effects on functional improvement, adherence, and retention compared to standard of care. We will calculate the cost per mental health patient achieving functional improvement and the incremental cost-effectiveness ratio (ICER) per new mental health diagnosis and DALY averted for each scale-up scenario. Analyses will be from the payer perspective, using economic productivity data to estimate mental health disability averted and incorporating costs of healthcare to evaluate economic impact.

## Discussion

This study is innovative in extending a breakthrough from the field of HIV to MH treatment, while also being the first to assess not only *whether* the SAIA implementation strategy works—but *how* it works—through testing causal pathway models. Systematic reviews of implementation strategies for mental health have found that nearly half produce no statistically significant effects on targeted implementation or clinical outcomes [90]. Leaders in the field have advocated that future studies testing implementation strategies specify strategy-mechanism-outcome linkages and test these mechanisms through causal pathway analyses [91]. Causal pathway analyses, and the specification of mechanisms of effect, are necessary to: (1) better understand why unsuccessful strategies fail; (2) inform the development of more effective and efficient implementation strategies; (3) identify mutable targets for future strategies; and (4) inform tailoring and matching strategies to barriers and contexts [91, 92]. Despite this urgent need in the field, a systematic review found that of 88 RCTs testing implementation strategies for mental health, zero met minimum criteria necessary for testing mediation hypotheses [92]. Mechanistic models of effect are needed to match/tailor strategies to barriers/contexts, prioritize strategy use, and expedite the development of more effective, efficient, and feasible strategies.

Applying the SAIA implementation strategy to task-shared MH systems optimization will distinguish between the “hard core” and “adaptable periphery” of the SAIA strategy, as well as its “transferability” to other disease areas and treatment cascades. Understanding which components of an implementation strategy are essential to effectiveness and which components may be adapted to suit context is critical to informing strategy scale-up. The present study provides a unique opportunity to distinguish between these components. By fixing the contextual determinants and modifying the application of SAIA, this study builds directly from prior studies and will directly inform which components are essential to effectiveness across disease areas [27, 29]. This project will also help generate an understanding of the overall “transferability” of the SAIA strategy to meet the needs of different treatment cascades or disease areas [93, 94]. Data on SAIA-MH effectiveness, determinants of implementation success, and mechanisms of effect (or non-effect) will be used to inform the use of SAIA across varied local contexts and complex treatment cascades. We believe that our focus on testing the multicomponent SAIA-MH strategy to optimize the linked MH cascade rather than a single intervention on a single siloed health indicator is innovative. Furthermore, the SAIA strategy has longevity because, rather than testing a single intervention that may become irrelevant after policy, system, or technology changes, SAIA-MH packages protocols and tools for data-driven systems optimization appropriate for the changing landscape of health delivery systems across LMICs.

Extending breakthroughs of implementation strategies for HIV treatment cascade optimization to MH care is an efficient and promising method for scientific advancement. The original “Systems Analysis and Improvement Approach” (SAIA) implementation strategy improved HIV treatment cascade performance and was accessible/user-friendly for frontline workers/managers [27, 29, 95]. In our preliminary developmental/exploratory project, SAIA-MH showed strong promise for mental health treatment cascade gains [25]. While pharmacological treatment for MH has system idiosyncrasies compared to HIV, both MH and HIV are chronic illnesses requiring screening/identification in multiple settings, referral to a centralized treatment center with information systems enabling longitudinal care, intra- and inter-facility referrals for pharmacy, laboratory, or other support services, and facility-community linkages to manage case-finding, medication management, and patient follow-up. For these reasons, proven strategies developed for the HIV cascade are ideal scientific building blocks for the development of strategies for MH treatment cascade optimization. If effective, the SAIA-MH implementation strategy has a large potential to be rapidly scaled-up to decrease gaps in task-shared MH treatments globally.

## Data Availability

Data emanating from this study will be submitted to the National Institute of Mental Health Data Archive (NDA) at https://nda.nih.gov/edit_collection.html?id=3898.

## Abbreviations

aOR: Adjusted odds ratio
CFIR: Consolidated Framework for Implementation Research
CI: Confidence interval
CQI: Continuous Quality Improvement
ERIC: Expert Recommendations for Implementation Change
FGDs: Focus Group Discussions
HIV: Human immunodeficiency virus
IDIs: In-depth interviews
LMIC: Low- and middle-income-countries
LTFU: Loss-to-follow-up
MH: Mental health
MHCAT: Mental Health Cascade Analysis Tool
RCT: Randomized controlled trial
SAIA-MH: Systems Analysis and Improvement Approach for Mental Health
SEM: Structural equation modeling
TB: Tuberculosis
WHODAS: World Health Organization Disability Assessment Scale
YLD: Years lived with disability
